# Sociodemographic characteristics associated with alcohol use among low-income Mexican older adults

**DOI:** 10.1186/s13011-016-0061-6

**Published:** 2016-04-29

**Authors:** Emma Aguila, Erick G. Guerrero, William A. Vega

**Affiliations:** Sol Price School of Public Policy, University of Southern California, 650 Childs Way, Los Angeles, CA 90089 CA USA; School of Social Work, University of Southern California, Los Angeles, CA USA; Preventive Medicine and School of Social Work, University of Southern California, Los Angeles, CA USA

**Keywords:** Drinking, Older adults, Mayan older adults, Mexico

## Abstract

**Background:**

Despite increasing concern about the quality of life of older adults, little is known about characteristics associated with health risk behaviors among older adults in middle-income countries. This study relied on unique longitudinal data to examine the relationship between sociodemographic characteristics and alcohol use among low-income older adults, one of the fastest-growing populations worldwide.

**Methods:**

This multilevel longitudinal analysis involved three waves of data (2008–2010) from 2,351 adults aged 70 or older in Yucatán, Mexico. Multilevel regressions models were used to test interactions among gender, speaking Mayan (indigenous language), and socioeconomic status to understand conditions associated with the odds of current alcohol use and the frequency and amount of alcohol use.

**Results:**

Half of the participants in this study report consuming alcohol in their lifetime, 21.58 % of whom were current alcohol users. Older adults reported consuming alcohol 1.15 days a week and 1.60 drinks per day. Speaking Mayan was associated with lower odds of current alcohol use. However, men who spoke Mayan reported higher odds of drinking alcohol compared to women and non-Mayan (Spanish) speakers. The positive relationship between socioeconomic status and alcohol use was also moderated by gender (male).

**Conclusions:**

Findings show that older and Mayan populations had lower odds of drinking in Yucatán, Mexico, whereas men were at highest risk of drinking alcohol, after adjusting for ethnic culture and socioeconomic status. Implications for health policy and epidemiological studies on substance use among older adults residing in low-income settings are discussed.

**Electronic supplementary material:**

The online version of this article (doi:10.1186/s13011-016-0061-6) contains supplementary material, which is available to authorized users.

## Background

Understanding alcohol use among older adults is critical because extended exposure to substances is associated with a higher likelihood of experiencing disability, cognitive impairment, noncommunicable diseases, falls, memory loss, and psychological and social isolation problems in old age [1]. Aging populations are expanding around the world [2] and there is increasing evidence that rates of alcohol use are rising among older populations, particularly in middle-income countries [3–5]. Yet there is little evidence of the individual characteristics associated with alcohol use among older adults in middle-income countries such as Mexico, where the older adult population is growing rapidly [6, 7].

Mexico has experienced significant growth in its population of individuals aged 70 years old or older. From 2014 to 2050, this population is expected to more than triple, from 5 million to 17 million, compared to an increase in total population from 119 million to 150 million [8]. Alcohol use is a major concern in Mexico. In 2011, 44.3 % of men and 19.7 % of women reported alcohol use during the previous month [9]. These estimates are based on the most authoritative source of information on alcohol use in the country, the Encuesta Nacional de Adicciones (National Survey of Addictions). However, these prevalence rates are limited to individuals between 12 and 65 years old from localities where no more than 50 % of inhabitants speak only an indigenous language [9].

There is limited systematic research that has provided statistical accounts of alcohol use among older adults from indigenous backgrounds [10]. However, studies have suggested that adult indigenous populations are more prone to consuming alcohol than nonindigenous populations in Mexico. Individuals in indigenous communities frequently include alcohol consumption as part of their daily lives [11] and use alcohol for various purposes, including providing traditional fermented beverages to small children to reduce hunger or help them relax [12–14].

Individuals with indigenous backgrounds account for more than 11 % of the older adult population in Mexico [15]. Indigenous populations are considered a vulnerable group in Mexico because their low income, language, and sociocultural background challenge their socioeconomic mobility. In 2012, the poverty rate among indigenous populations (whose members speak an indigenous language) was almost twice (76.8 %) that of nonindigenous populations (43.0 %). For the same year, the extreme poverty rate of indigenous populations was 38.0 %, compared to 7.9 % for nonindigenous individuals [16].

A large literature has indicated that socioeconomic status (SES) plays a role in individuals’ alcohol use patterns in the United States. However, there is limited evidence of this phenomenon among older adults in Mexico. The available evidence on the relationship between proxies for SES and alcohol use is mixed. For example, Aguilar-Navarro et al. [6] found that higher levels of education were associated with alcohol use among Mexican older adults. Similarly, Borges et al. [7] studied an urban sample of adults 70 or older in Mexico City and found that having at least a high school education was associated with alcohol use. These findings suggest relationships between alcohol use and gender, SES, and indigenous background, but the direction is not clear.

Although gender is a robust indicator of likelihood and amount of alcohol use, with Latino men showing a higher propensity to drink alcohol across subgroups based on country of origin [17–19], there is limited understanding of the interacting roles of gender and indigenous background (e.g., Mayan) and gender and SES (income and education) regarding alcohol use. There is evidence of traditional sex roles among indigenous populations in Mexico. Indigenous women generally are dedicated to housekeeping and taking care of children, assume a submissive role with respect to men, have less access to education than men, and are not allowed to receive money or in-kind inheritances [20–23], which may imply similar differences in alcohol use between indigenous men and women as in the general population.

In this study, we sought to obtain preliminary evidence of the characteristics of alcohol use in an underresearched population in Mexico, older indigenous adults who speak Mayan. This is considered one of the two largest groups of older indigenous adults in Mexico [21]. We used a rich three-wave longitudinal dataset equivalent to the U.S. Health and Retirement Survey (HRS) and Mexican Health and Aging Survey collected between 2008 and 2010 to analyze a supplemental income program for adults aged 70 or older in the state of Yucatán, Mexico. Yucatán is a key region to explore differences between Mayan and non-Mayan older adult populations because this state has the highest proportion of indigenous households (51.4 %) in Mexico [22]. Thus, our exploratory hypotheses were as follows.

### Hypothesis 1

Compared with women and non-Mayan speakers, men who speak Mayan will be associated with a higher likelihood of current alcohol use.

### Hypothesis 2

Compared with women and individuals with lower SES, men with higher SES will be associated with a higher likelihood of current alcohol use.

## Methods

### Sampling frame

Participants were recruited for a regional study in Yucatán, Mexico, to analyze the effects of a supplemental income program using a randomized design at the town level [24]. All households in the selected communities—Valladolid had 45,868 inhabitants and Motul had 21,508 inhabitants [25]—were screened to identify households with adults aged 70 or older. The study design, recruitment and follow-up procedures, and inclusion and exclusion criteria have been described in greater detail elsewhere [24]. The response rate was 93.3 % for Wave 1 (August to November 2008), 84.9 % for Wave 2 (June and July 2009), and 79.5 % for Wave 3 (June and July 2010). Response rates were computed using American Association for Public Opinion Research [26] guidelines. The survey involved an in-person interview to collect information on socioeconomic, demographic, and household characteristics; health status; health risk behaviors; other self-reported health indicators; anthropometrics; performance tests; and biomarkers of blood pressure, lung capacity, and grip strength. The survey instrument was drawn from the HRS, whose measures were translated, validated, and tested in the Mexican Health and Aging Survey for the Mexican context. The final sample consisted of 2,351 individuals aged 70 or older with observations from 2008 to 2010. The study protocol was approved by the Internal Review Board of the RAND Corporation (Protocol approval number 2008-0513-CR07). A written informed consent according to the Helsinki declaration II was obtained from the study participants. To obtain written informed consent to participate in the survey, we followed standard and internationally applied protocols from the HRS [27]. For instance, when participants were unable to provide her/his signature for the written informed consent (e.g., due to low literacy), the interviewer explained the study, answered questions and addressed concerns before obtaining oral consent and signing the consent form as a “witness to informed consent”.

### Measures

#### Dependent variables

To assess alcohol consumption, we used four self-reported items: (a) lifetime use of alcohol (1 = *ever drank alcohol*, 0 = *nondrinker*); (b) current use of alcohol (1 = *current alcohol user*, 0 = *nondrinker*); (c) number of days of alcohol use per week during the previous month; and (d) number of drinks per day on days of alcohol use during the previous month. We also calculated the number of respondents who used alcohol two or more days per week during the previous month and the number of respondents who had three or more alcoholic drinks per day on days of alcohol use during the previous month [28–32]. The two latter measures, number of days of alcohol use and number of drinks per day, were skewed at 1 day (72.22 %) and two drinks (77.77 %), respectively. Hence, we created two dichotomous outcome measures (see Table 1): the likelihood of drinking 2 or more days per week (27.77 %) and having three or more drinks per week (22.22 %). To support this threshold, we also considered the criteria for problematic alcohol use for older adult populations suggested by the National Institute of Alcohol Abuse and Alcoholism [33] of one standard drink per day or seven standard drinks per week and no more than three drinks on one occasion.

**Table 1:** Descriptive statistics

	Wave 1 % or M (SD)	Wave 2 % or M (SD)	Wave 3 % or M (SD)	Response format
Dependent variables				
Alcohol use				
Lifetime use	54.47	60.68	66.58	Ever drank alcohol
Current use	11.74	10.50	9.88	Currently drinking alcohol
Days of use per week	1.15 (1.38)	0.98 (1.40)	0.87 (1.32)	Days of use per week during previous month
Use 2+ days per week	27.77	21.13	20.00	Alcohol use twice or more per week
Drinks per day	1.60 (2.12)	1.58 (2.37)	1.14 (1.74)	Alcoholic drinks per day of use
3+ drinks per day	22.22	23.40	14.87	Three or more drinks per day
Independent variables				
Primary language				
Mayan	36.70	37.71	38.02	Speak Mayan as primary language
Gender				
Male	48.06	47.52	47.15	Male gender
Socioeconomic status				
Education				
0–2 years	64.82	67.15	67.55	0 to 2 years of formal education
3+ years	33.56	32.62	31.17	3 or more years of formal education
Household income				
Monthly income, MXN	1,322.50 (2,986.69)	1,760.80 (2,462.66)	1,509.23 (1,662.57)	Mean monthly household income in constant pesos
Tertile 1 or 2 (T1 or T2)	66.18	62.70	60.97	Household income tertile 1 or 2
Tertile 3 (T3)	31.26	31.21	30.06	Household income tertile 3
Control variables				
Age	77.95 (6.45)	78.35 (6.36)	78.81 (6.37)	Age in years
70–74	37.39	34.59	29.97	70 to 74 years old
75–79	28.43	30.04	31.83	75 to 79 years old
80–84	17.29	17.16	18.33	80 to 84 years old
85+	16.90	18.21	19.87	85 years old or older
Marital status				
Married or partner	52.19	51.93	50.03	Married or living with partner
Household size				
1	13.31	12.36	11.36	1 resident
2–4	60.87	59.06	58.68	2 to 4 residents
5–7	20.54	22.31	23.90	5 to 7 residents
8+	5.27	6.27	6.05	8 or more residents
Self-reported health				
Good, very good, or excellent	17.86	24.58	25.17	Good, very good, or excellent self-rated health
Fair	61.38	62.34	61.60	Fair self-rated health
Poor	20.50	13.04	13.06	Poor self-rated health
Health indicators				
Depression	0.84 (2.01)	0.46 (1.49)	0.29 (1.19)	CIDI-SF depression score, range = 0–7
Lifetime liver or kidney infection	6.35	9.00	9.69	Diagnosed with liver or kidney infection
Current tobacco use	3.45	3.05	2.66	Currently smoke cigarette
Health insurance	72.05	76.33	79.50	Public or private health insurance
Observations	2,351	2,201	1,883	Number of respondents from baseline

*Note. CIDI-SF* Composite International Diagnostic Interview Short-Form, *MXN* Mexican peso

#### Independent variables

We included a binary variable for primary language spoken (1 = *Mayan*, 0 = *Spanish*) using survey data that indicated the language used during the screening process and interviews. This dataset is particularly suitable to analyzing indigenous population because the survey instrument was translated to Spanish and Mayan and the interviews were conducted in Spanish or Mayan. At least half of the interviewers were bilingual (Spanish and Mayan) [24]. Interviews were conducted in Mayan as opposed to other indigenous languages because according to a census in 2010, 98.6 % of the individuals who spoke an indigenous language in the state of Yucatán spoke Mayan [34]. In this sample, only 0.8 % of participants reported speaking an indigenous language other than Mayan. We also included gender (1 = *male*, 0 = *female*), SES measured as years of education (*0–2* or *3 or more*), and household monthly income divided by tertiles. We defined household income as the sum of all wages, income from businesses or farms, pensions, income from properties, capital assets income (including income from checking and savings accounts, fixed investments, stocks, bonds, and shares), and transfers, both monetary and in-kind, from relatives, friends, and governmental institutions. We deflated household income with the Mexican National Consumer Price Index base year of the last 2 weeks of December 2010 [35]. We present income data in Mexican pesos and equivalents in U.S. dollars based on purchasing power parity (PPP). Because we made comparisons between years, U.S. PPP dollars were adjusted to compensate for inflation. Therefore, all figures are expressed in 2014 U.S. PPP dollars [36].

We also included interaction terms between gender and speaking Mayan as a primary language and between gender and SES. To develop the interaction of gender and SES, we transformed SES into two dichotomous variables: (a) 3 or more years of education and (b) income in the third tertile. These two dummy variables were used to create a dichotomous variable in which 3 years or more of education and third-tertile income represent the multilevel definition of SES.

#### Control variables

We controlled for the following variables: age (70–74, 75–79, 80–84, and 85 or older); number of household residents (1, 2–4, 5–7, and 8 or more); health indicators measured by self-reported health status (5-point scale: 0 = *excellent*, 1 = *very good*, 2 = *good*, 3 = *fair*, and 4 = *poor*) and divided into three categories: (a) excellent, very good, and good, (b) fair, and (c) poor; a self-reported dummy variable of liver or kidney infection based on diagnosis by a doctor or medical personnel; and a depression score (ranging from 1–7) from the Composite International Diagnostic Interview Short-Form from the HRS. The latter instrument evaluates major depressive episodes (feeling depressed, tired or having low energy, appetite change, trouble sleeping, trouble concentrating, feeling worthless, and thinking about death) as defined by the revised third edition of the *Diagnostic and Statistical Manual of Mental Disorders* of the American Psychiatric Association [37]. Additionally, we include a binary variable for health insurance (1 = *public or private health insurance*, 0 = *none*).

### Statistical analysis

We relied on Stata version 14 to conduct multilevel (measures nested in three waves) logistic regression models for dichotomous variables (current alcohol use, more than two alcoholic drinks per week, and more than three drinks per day) [38, 39]. We used the XTLOGIT command to model our binary outcome (alcohol use and frequency of alcohol use with a binary indicator of more than two alcoholic drinks per week and more than three drinks per day) [40, 41]. To reduce sample selection bias, we also conducted three main robustness analyses: (a) we compared the first-wave characteristics of individuals who died prior to subsequent waves with those who remained alive to assess the effects of mortality bias in this study; (b) we compared the first-wave characteristics of individuals who did not complete subsequent waves with longitudinal respondents to assess potential sample attrition bias; and (c) we analyzed item nonresponse per variable in the analysis to assess the quality of the data and any other potential source of bias.

## Results

Regarding alcohol use, 54.47 % of older adults reported consuming alcohol in their lifetime, 21.58 % of whom were current alcohol users. By gender and language, 73.60 % of men (78.34 % non-Mayan and 65.97 % Mayan speakers) and 36.75 % of women (42.89 % non-Mayan and 25.52 % Mayan speakers) reported consuming alcohol in their lifetime; 21.54 % of men (22.89 % non-Mayan and 18.95 % Mayan speakers) and 21.65 % of women (23.37 % non-Mayan and 16.36 % Mayan speakers) were current alcohol users. On average, older adults reported consuming alcohol 1.15 days a week and 1.60 drinks per day. In our sample, 6.16 % of men and 0.66 % of women exceeded the suggested standard for older adults of one drink a day or seven drinks a week, with no more than three drinks on one occasion [33]. Five percent of men and 0.49 % of women exceeded the daily limit and 4.23 % of men and 0.58 % of women exceeded the weekly limit (results not shown).

### Hypothesis testing

Hypothesis 1 was supported. Compared to women and Spanish speakers, men and Mayan speakers had a higher likelihood of current alcohol use (*OR* = 3.206, 95 % CI = 1.649, 6.233). See results in Table 2.

**Table 2:** Multilevel logistic regression of alcohol use

	Current alcohol use			2+ Days of use per week			3+ drinks per day		
	*OR*	*SE*	95 % CI	*OR*	*SE*	95 % CI	*OR*	*SE*	95 % CI
Mayan language	0.333***	0.058	0.101, 0.287	0.418	0.201	0.163, 1.073	0.499	0.248	0.188, 1.322
Male	1.036	0.182	0.735, 1.461	2.008**	0.501	1.231, 3.275	0.610	0.167	0.357, 1.043
Socioeconomic status									
3+ years of education	1.136	0.181	0.831, 1.552	1.178	0.271	0.750, 1.850	0.757	0.182	0.472, 1.213
Household income T3	1.043	0.164	0.767, 1.418	1.188	0.291	0.735, 1.921	1.613	0.422	0.966, 2.693
Interaction terms									
Mayan × male	3.206***	1.088	1.649, 6.233	1.673	0.937	0.558, 5.014	0.907	0.528	0.290, 2.838
Male × 3+ educ. × T3 inc.	1.708*	0.467	1.000, 2.919	1.314	0.497	0.626, 2.757	1.040	0.408	0.481, 2.245
Age									
75–79	0.674**	0.102	0.502, 0.906	0.794	0.172	0.520, 1.213	0.816	0.184	0.524, 1.268
80–84	0.400***	0.084	0.265, 0.603	0.762	0.238	0.413, 1.404	0.407**	0.141	0.206, 0.804
85+	0.205***	0.055	0.121, 0.348	0.525	0.224	0.227, 1.210	0.290*	0.140	0.113, 0.745
Household size									
2–4	0.321***	0.058	0.226, 0.457	0.697	0.179	0.422, 1.151	0.610	0.167	0.357, 1.043
5–7	0.365***	0.079	0.238, 0.559	0.594	0.183	0.325, 1.085	0.790	0.249	0.425, 1.467
8+	0.147***	0.060	0.066, 0.326	0.295	0.195	0.081, 1.075	0.465	0.296	0.134, 1.617
Self-reported health									
Fair	0.425***	0.056	0.328, 0.551	0.527**	0.111	0.349, 0.795	0.682	0.143	0.452, 1.029
Poor	0.233***	0.052	0.150, 0.362	0.679	0.262	0.319, 1.445	0.663	0.275	0.295, 1.494
Depression	0.954	0.040	0.879, 1.035	1.017	0.069	0.890, 1.163	0.948	0.071	0.819, 1.098
Lifetime liver or kidney infection	5.782***	1.808	3.133, 10.671	0.939	0.313	0.488, 1.805	1.521	0.517	0.780, 2.963
Health insurance	0.574***	0.086	0.427, 0.771	--	--	--	0.817	0.200	0.506, 1.320
Current tobacco use	3.206***	1.088	1.649, 6.233	1.673	0.937	0.558, 5.014	1.521	0.517	0.290, 2.838
Wave									
2	0.659***	0.083	0.514, 0.843	0.709	0.154	0.463, 1.085	1.355	0.303	0.875, 2.101
3	0.623***	0.088	0.473, 0.820	0.815	0.197	0.507, 1.310	0.705	0.181	0.426, 1.165
Observations	5,066			616			616		
Log likelihood	-1,686.430			-381.734			-376.090		
Chi-square	537.20			41.12			51.61		

*Note.* Figures represent exponentiated coefficients. The full sample was analyzed for current alcohol use, whereas only current alcohol users were considered for the two other outcomes. Reference categories were 70–74 years old for age; one resident for household size; good, very good, or excellent for self-rated health; and Wave 1 for wave. Male × 3+ educ. × T3 inc. is the interaction between gender and SES (3 or more years of education and income in the third tertile). *CI* confidence interval, *OR* odds ratio, *SE* standard error

*p < .05. **p < .01. ***p < .001

Hypothesis 2 was supported. Compared with women and individuals with lower SES, men with higher SES were associated with a higher likelihood of current alcohol use (*OR* = 1.708, 95 % CI = 1.000, 2.919).

Older adults who spoke Mayan had reduced odds of current use of alcohol compared with non-Mayan-speaking older adults (*OR* = 0.333, 95 % CI = 0.101, 0.287). Men had increased odds of 2+ days of alcohol use per week (*OR* = 2.008, 95 % CI = 1.231, 3.275). But, findings relative to frequency and amount of alcohol consumed were not statistically significant overall.

Although not hypothesized in this study, other individual characteristics were associated with use of alcohol among Mexican older adults (see Table 2). Regarding alcohol use, compared with the youngest group, the oldest group (85 years old or older) had lower odds of current alcohol use. Similarly, having more household residents, self-reported fair and poor health, and health insurance were associated with lower odds of current alcohol use. Older adults who reported current tobacco use had higher odds of current alcohol use.

## Discussion

This exploratory study sought to identify the relationship between sociodemographic characteristics of Mexican low-income older adults and use of alcohol. We found that the roles of gender and Mayan background are critical in understanding alcohol use in this sample. Current use of alcohol and frequency of use were much higher among men. But frequency (1.15 days a week) and amount (1.60 drinks per day) were relatively lower in this sample compared to rates reported for the general Mexican adult population [9]. Overall, findings are consistent with other studies and conceptual frameworks that highlight male socialization as a risk factor for alcohol use [42–45].

The significant role of gender was also seen when considering culture and SES. We found significant interactions between men who spoke Mayan and current alcohol use. Although the amount of use was not statistically significant, the higher likelihood of use among older adult men with Mayan backgrounds is a major finding. Although older adult Mayan speakers as a population had lower rates of use and thus lower risk of current alcohol use than non-Mayan speakers, male Mayan speakers had higher odds of current alcohol use compared to women and non-Mayan speakers. This may be explained by our conceptual framework that highlights the traditional role of women with regard to not engaging in risk-taking behaviors; in this case, the most traditional group, women who spoke Mayan, reported the lowest rates of current alcohol use.

SES (education and income) also interacted with gender. Men with higher SES reported a higher likelihood of current alcohol use. This finding provides preliminary evidence that income may have a significant effect beyond that of male gender in this population of older adults.

We also found that 54.47 % of participants reported consuming alcohol in their lifetime. These results are comparable to Borges et al.’s [7] study, in which 63.5 % of the sample (adults aged 69 or older in Mexico City) reported ever using alcohol. It is also comparable to alcohol use in middle-income countries; e.g., 42.0 % of older adults in Ghana (nationally representative sample of adults aged 50 or older) reported drinking alcohol in their lifetime [46].

In this study, we found 21.58 % of participants who ever drank alcohol were current alcohol users, including 21.54 % of men and 21.65 % of women. Other studies reporting drinking patterns by Latin American regions found that among adults 65 years old or older, 27.6 % of women and 22.1 % of men in the Andean region, 26.7 % of women and 45.5 % of men in the Central region, and 51.6 % of women and 69.6 of men in the Southern region were current drinkers [47].

Finally, it is important to note that the youngest, healthiest, and least-resourced (in terms of income and having fewer residents in the home) older adults had the highest risk of alcohol use in this study. Although evidence suggests that SES and speaking Mayan was associated with drinking, these results were driven by male gender. Albeit conjectural, these findings may also point to the overall resilience of the indigenous population in this region.

### Limitations

Limitations of the present study should be noted. First, some of the measures were cross-sectional, preventing us from establishing causality. However, analysis of three waves of data provided robust estimates. Second, outcome measures of alcohol use are susceptible to underreporting the actual prevalence of these outcomes. However, data collectors were trained to reduce underreporting by using follow-up questions regarding situations in which participants used alcohol.

Another limitation is the risk of sample selection bias. To reduce this risk, we conducted several robustness checks and did not find statistically significant differences in terms of alcohol outcomes. This may indicate no sample selection bias in terms of alcohol use between participants who died and those who completed all three assessments (see Additional file 1: Table S1). Likewise, we found no differences between individuals who dropped out and panel respondents in terms of lifetime use and current use of alcohol (see Additional file 2: Table S2). We found differences among drinkers in terms of the number of days of alcohol use and drinks per day, with individuals who dropped out reporting higher frequency of use. However, the number of individuals lost to attrition (27 drinkers) was too small to preclude any potential sample selection bias.

Finally, our analyses only allowed us to generalize findings about alcohol use to the sampling frame and not to other regions. Nonetheless, this issue was somewhat mitigated by the large sample of older adults, random selection procedures, and use of three-wave data. We believe that the estimates obtained are robust and findings from this sample are unique, providing preliminary data on a growing population segment in Mexico.

## Conclusion

Given the limited (almost nonexistent) information on alcohol use among older adults in Mexico, the current study provides important information for future epidemiological studies on substance use considering key differences based on gender, culture, and SES. The aging of the Mexican population has been noted as a consequential demographic transition for the country’s health care system [48]. Because the older population in Mexico is expected to grow exponentially, as in many other middle-income countries [48], effective health care and public health policies are needed to manage the population disease burden and moderate public expenditures in an era of expanding longevity. In particular, future research should consider the interaction between gender, culture and SES when exploring healthy aging among low income populations in Mexico.

### Ethics, consent, and permissions

This study was reviewed and approved by the Institutional Review Board at RAND Corporation (Protocol approval number 2008-0513-CR07). The principal investigator, also the corresponding author, has obtained consent to publish from the participants in the study (residents of Yucatán, Mexico).

## Additional files

Additional file 1: Table S1. Wave 1 Characteristics Deceased and Alive (DOC 54 kb) Additional file 2: Table S2. Wave 1 Characteristics Attriters and Panel (DOC 54 kb)
