# Supplementary material for: Sociodemographic characteristics associated with alcohol use among low-income Mexican older adults
Source: Subst Abuse Treat Prev Policy. 2016 Apr 29;11:16. doi: 10.1186/s13011-016-0061-6 (PMC4850697; doi:10.1186/s13011-016-0061-6)
Supplement: Additional file 1: Table S1. — Wave 1 Characteristics Deceased and Alive (DOC 54 kb) [file 13011_2016_61_MOESM1_ESM.doc]

| Additional file 1: Table S1. *Wave 1 Characteristics Deceased and Alive* | | | |
| --- | --- | --- | --- |
|  | Deceased | Alive | Δ |
| Outcomes of interest |  |  |  |
| Alcohol use |  |  |  |
| Lifetime use, % | 51.87 | 54.77 | 2.90 |
| Current use, % | 7.44 | 12.23 | 4.80*** |
| Days alcohol per week (Dec. *n* = 22, alive *n* = 417) | 1.32 | 1.15 | -0.17 |
| Drinks per day (Dec. *n* = 21, alive *n* = 419) | 1.62 | 1.59 | -0.02 |
| Independent variables |  |  |  |
| Primary language |  |  |  |
| Mayan, % | 33.88 | 37.03 | 3.15 |
| Gender |  |  |  |
| Male, % | 47.52 | 48.13 | 0.61 |
| Socioeconomic status |  |  |  |
| Education |  |  |  |
| 3 years or more of education (%) | 32.23 | 33.71 | 1.48 |
| Household income |  |  |  |
| Household monthly income (mean, MXN) | 1,099.55 | 1,347.99 | 248.45 |
| Household monthly income, tertile 3, % | 27.69 | 31.67 | 3.99 |
| Control variables |  |  |  |
| Age (mean) | 82.33 | 77.45 | -4.88*** |
| 70-74, % | 14.88 | 39.98 | 25.10*** |
| 75-79, % | 25.62 | 28.75 | 3.13 |
| 80-84, % | 21.07 | 16.85 | -4.23 |
| 85+, % | 38.43 | 14.42 | -24.01*** |
| Marital status |  |  |  |
| Married or couple, % | 43.80 | 53.15 | 9.35** |
| Household size |  |  |  |
| 1 household resident, % | 8.68 | 13.85 | 5.17** |
| 2-4 household residents, % | 60.33 | 60.93 | 0.60 |
| 5-7 household residents, % | 24.38 | 20.10 | -4.28 |
| 8 or more household residents, % | 6.61 | 5.12 | -1.49 |
| Self-reported health status |  |  |  |
| Good, very good, or excellent, % | 12.40 | 18.49 | 6.10** |
| Fair, % | 56.61 | 61.93 | 5.31 |
| Poor, % | 30.58 | 19.35 | -11.23*** |
| Other health indicators |  |  |  |
| CIDI-SF depression score (0-7) | 0.77 | 0.84 | 0.08 |
| Lifetime liver or kidney infection, % | 8.26 | 6.13 | -2.14 |
| Current tobacco use, % | 4.07 | 3.31 | -0.76 |
| Health insurance, % | 72.31 | 72.02 | -0.29 |
| Observations | 242 | 2,109 |  |
| *Note.* Dec. = deceased. **p* < .05, ***p* < .01, ****p* < .001. | | | |
