# Supplementary material for: Sociodemographic characteristics associated with alcohol use among low-income Mexican older adults
Source: Subst Abuse Treat Prev Policy. 2016 Apr 29;11:16. doi: 10.1186/s13011-016-0061-6 (PMC4850697; doi:10.1186/s13011-016-0061-6)
Supplement: Additional file 2: Table S2. — Wave 1 Characteristics Attriters and Panel (DOC 54 kb) [file 13011_2016_61_MOESM2_ESM.doc]

| Additional file 2: Table S2. *Wave 1 Characteristics Attriters and Panel* | | | |
| --- | --- | --- | --- |
|  | Attriters | Panel | Δ |
| Outcomes of interest |  |  |  |
| Alcohol use |  |  |  |
| Lifetime use, % | 53.39 | 55.43 | 2.04 |
| Current use, % | 12.22 | 11.97 | -0.25 |
| Days alcohol per week (Attr. *n* = 27, Panel *n* = 338) | 2.19 | 1.02 | -1.16** |
| Drinks per day (Attr. *n* = 27, Panel *n* = 340) | 2.89 | 1.46 | -1.43* |
| Independent variables |  |  |  |
| Primary language |  |  |  |
| Mayan, % | 25.34 | 40.15 | 14.81*** |
| Gender |  |  |  |
| Male, % | 47.51 | 47.94 | 0.43 |
| Socioeconomic status |  |  |  |
| Education |  |  |  |
| 3 years or more of education (%) | 38.91 | 31.98 | -6.93*** |
| Household income |  |  |  |
| Household monthly income (mean, MXN) | 1,552.63 | 1,311.05 | -241.58 |
| Household monthly income, tertile 3, % | 33.48 | 32.11 | -1.38 |
| Control variables |  |  |  |
| Age (mean) | 77.77 | 77.45 | -0.32 |
| 70-74, % | 38.79 | 39.78 | 0.99 |
| 75-79, % | 27.57 | 29.24 | 1.67 |
| 80-84, % | 17.76 | 16.21 | -1.55 |
| 85+, % | 15.89 | 14.78 | -1.11 |
| Marital status |  |  |  |
| Married or couple, % | 55.66 | 52.56 | -3.10 |
| Household size |  |  |  |
| 1 household resident, % | 14.03 | 13.28 | -0.75 |
| 2-4 household residents, % | 66.06 | 60.72 | -5.34 |
| 5-7 household residents, % | 14.03 | 20.95 | 6.92** |
| 8 or more household residents, % | 5.88 | 5.05 | -0.83 |
| Self-reported health status |  |  |  |
| Good, very good, or excellent, % | 24.89 | 17.52 | -7.37* |
| Fair, % | 57.47 | 62.72 | 5.25 |
| Poor, % | 17.65 | 19.51 | 1.87 |
| Other health indicators |  |  |  |
| CIDI-SF depression score (0-7) | 0.69 | 0.91 | 0.22 |
| Lifetime liver or kidney infection, % | 6.33 | 5.93 | -0.40 |
| Current use, % | 4.07 | 3.31 | -0.76 |
| Health insurance, % | 66.52 | 73.44 | 6.93* |
| Observations | 221 | 1,604 |  |
| Note. Att. = attriters. **p* < .05, ***p* < .01, ****p* < .001. | | | |
